# The association of circadian parameters and the clustering of fatigue, depression, and sleep problems in breast cancer survivors: a latent class analysis

**DOI:** 10.1007/s11764-022-01189-w

**Published:** 2022-03-23

**Authors:** Belle H. de Rooij, Imogen Ramsey, Felix J. Clouth, Nadia Corsini, Jane S. Heyworth, Brigid M. Lynch, Jeff K. Vallance, Terry Boyle

**Affiliations:** 1 Netherlands Comprehensive Cancer Organisation, Utrecht, The Netherlands; 2 CoRPS - Center of Research On Psychology in Somatic Diseases, Department of Medical and Clinical Psychology, Tilburg University, Tilburg, The Netherlands; 3 Rosemary Bryant AO Research Centre, UniSA Clinical and Health Sciences, University of South Australia, Adelaide, South Australia Australia; 4 Department of Methodology and Statistics, Tilburg University, Tilburg, The Netherlands; 5 School of Population and Global Health, The University of Western Australia, Perth, WA Australia; 6 Cancer Epidemiology Division, Cancer Council Victoria, Melbourne, VIC Australia; 7 Centre for Epidemiology and Biostatistics, Melbourne School of Population and Global Health, University of Melbourne, Melbourne, Australia; 8 Faculty of Health Disciplines, Athabasca University, Athabasca, Canada; 9 Australian Centre for Precision Health, Allied Health and Human Performance, University of South Australia Cancer Research Institute, Adelaide, South Australia Australia

**Keywords:** Circadian rhythm, Breast cancer, Fatigue, Depression, Insomnia

## Abstract

**Purpose:**

Circadian rhythms control a wide range of physiological processes and may be associated with fatigue, depression, and sleep problems. We aimed to identify subgroups of breast cancer survivors based on symptoms of fatigue, insomnia, and depression; and assess whether circadian parameters (i.e., chronotype, amplitude, and stability) were associated with these subgroups over time.

**Methods:**

Among breast cancer survivors, usual circadian parameters were assessed at 3–4 months after diagnosis (T0), and symptoms of fatigue, depression, and insomnia were assessed after 2–3 years (T1, *N* = 265) and 6–8 years (T2, *N* = 169). We applied latent class analysis to classify survivors in unobserved groups (“classes”) based on symptoms at T1. The impact of each of the circadian parameters on class allocation was assessed using multinomial logistic regression analysis, and changes in class allocation from T1 to T2 using latent transition models.

**Results:**

We identified 3 latent classes of symptom burden: low (38%), moderate (41%), and high (21%). Survivors with a late chronotype (“evening types”) or low circadian amplitude (“languid types”) were more likely to have moderate or high symptom burden compared to “morning types” and “vigorous types,” respectively. The majority of survivors with moderate (59%) or high (64%) symptom burden at T1 had persistent symptom burden at T2.

**Implications for Cancer Survivors:**

A late chronotype and lower circadian amplitude after breast cancer diagnosis were associated with greater symptoms of fatigue, depression, and insomnia at follow-up. These circadian parameters may potentially be novel targets in interventions aimed at alleviating symptom burden among breast cancer survivors.

**Supplementary Information:**

The online version contains supplementary material available at 10.1007/s11764-022-01189-w.

## Introduction

Breast cancer survivors report a multiplicity of symptoms that significantly and persistently impair their quality of life [1]. To date, symptoms in cancer survivors have mostly been studied in isolation, even though many symptoms share a common biological cause [2]. For instance, some behavioral symptoms, including fatigue, depression, and sleep problems, are likely triggered by common inflammatory and/or neuroendocrine responses to the cancer and/or treatments [3]. As a result, fatigue, depression, and insomnia often co-occur in the same individual; a phenomenon called symptom-clustering [4, 5]. Research into the underlying mechanisms of symptom-clustering is still in its infancy [6].

Misalignment of circadian (24-h) physiological processes, including hormone secretion, body temperature, and sleep–wake cycles, is a suspected risk factor for developing breast cancer [7]. The cancer itself [8] or breast cancer treatments [9] may cause further disruptions to circadian rhythms. Important parameters of the circadian rhythm are *chronotype* (i.e., preference for early or late wake- and bed-time), *amplitude* (i.e., ability to overcome drowsiness), and *stability* (i.e., affinity for routine sleeping) [10]. Compared with healthy controls, breast cancer survivors have relatively flattened 24-h production of the stress hormone cortisol with elevated levels in the evening [8, 11], a pattern that often results in a delayed circadian phase and sleep–wake cycle [12]. The impact of disrupted sleep schedules (i.e., due to shift-work) on sleepiness appears to be greater in individuals with low amplitude and high stability of their circadian cycles [13].

Evidence suggests that in healthy populations, individuals with a late chronotype (“evening type”), low amplitude (“languid type”), or instability (“flexible type”) of circadian cycles have increased risks of depression [14], fatigue [15], and poorer sleep quality and duration [13]. Therefore, circadian preference may influence the prevalence and clustering of these symptoms in breast cancer survivors. Insights into the underlying pathophysiological mechanisms of the depression, fatigue, and insomnia symptom cluster may provide directions for research to determine whether these circadian preferences can be potentially modified using psycho-oncological or pharmaceutical interventions such as timed bright light exposure [16] or melatonin supplements [17]. Therefore, in this study, we aimed to (1) identify groups of breast cancer survivors based on symptoms of fatigue, depression, and insomnia, and 2) assess whether circadian parameters (i.e., chronotype, amplitude, and stability) at baseline are associated with symptom burden at follow-up.

## Methods

### Design

The current study includes breast cancer survivors living in Western Australia (WA) who participated in the Breast Cancer Employment and Environment Study (BCEES) between 2009 and 2011 (referred to as “T0” henceforth) [18], and a subsequent study (Accurate Measurement of Physical Activity and Sedentary Time Among Breast Cancer Survivors Study; ACCEL-Breast) in 2013 [19] (referred to as “T1” henceforth) and 2017 (referred to as “T2” henceforth).

### Population

For BCEES, all women diagnosed with a first incident invasive breast cancer between May 2009 and January 2011 were identified through the Western Australian Cancer Registry, of whom 2084 were deemed eligible (Supplementary file 1). Eligibility criteria included being female, 18 to 80 years of age and living in WA at the time of diagnosis, not having any serious other illness, and understanding English. From the 1205 women who completed the survey at T0 (57.8% response fraction), the 600 most recently diagnosed breast cancer survivors were invited to participate in the ACCEL-Breast study between April and December 2013 (T1). Survivors who participated at T1 were subsequently invited to complete a follow-up questionnaire between August and October 2017 (T2).

### Measures

Clinical variables including time since diagnosis and cancer stage at diagnosis were derived from the Western Australian Cancer Registry. Menopausal status, smoking status, and BMI at diagnosis were self-reported at T0 and education, ethnicity, marital status, employment status, comorbidities, and cancer treatments received were self-reported in the follow-up questionnaire at T1.

Chronotype (i.e., morningness/eveningness), circadian amplitude (i.e., languidness/vigorousness), and circadian stability (i.e., flexibility/rigidity) were assessed at T0. *Chronotype* was assessed using the Horne-Ostberg Morningness/Eveningness scale [20], consisting of 14 items scored on a 4-point ordinal scale, such as follows: “How easy do you find it to get up in the morning?” (“very difficult” to “very easy”) and 5 items scored on along a continuum of timeslots, such as “What time would you get up/ go to bed if you were entirely free to plan you day?” (“5:00–6:30 AM” to “11AM–noon”). Total scores can range from 16 to 86, with lower scores indicating a higher degree of eveningness and higher scores indicating a higher degree of morningness. The internal consistency in our sample was good (Cronbach’s alpha = 0.77). Circadian *amplitude* and *stability* were assessed using the Circadian Type Inventory [21], consisting of 11 items scored on a 5-point Likert scale (“almost never” to “almost always”). The amplitude scale (5 items) ranges from 5 to 25, with lower scores indicating a higher degree of vigorousness and higher scores indicating a higher degree of languidness (Cronbach’s alpha = 0.73). The stability scale (6 items) ranges from 6 to 30, with lower scores indicating more rigid habits and higher scores indicating more flexibility (Cronbach’s alpha = 0.80). To obtain equal group sizes, survivors were categorized into tertiles, separately for each circadian parameter: chronotype (“morning type” (> 64), “neither type” (57–64), and “evening type” (< 57)); circadian amplitude (“vigorous type” (< 13), “neither type” (13–16), and “languid type” (> 16)); and circadian stability (“rigid type” (< 11), “neither type” (11–15), and “flexible type” (> 15)).

Symptoms of fatigue, insomnia, and depression were measured at T1 and T2. *Fatigue* was assessed using the 13-item FACIT-Fatigue [22]. Items were scored on a 5-point Likert scale (“not at all” to “very much”), with higher scores indicating more fatigue, except for two items that were reversed (“I have energy” and “I am able to do my usual activities”). *Depression* was measured with the 9-item Patient Health Questionnaire (PHQ9) [23]. Items were scored on a 4-point Likert scale (“not at all” to “nearly every day”), with higher scores indicating that more depression *Sleep quality* was measured with the Pittsburgh Sleep Quality Index (PSQI) [24], consisting of 4 open questions about wake- and bed-time, hours of sleep, and minutes to fall asleep, and 14 questions scored on 4-point Likert scales with higher scores indicating more sleep problems. Seven component scores were calculated, as described previously [24].

Items from the PHQ9 that overlapped with the FACIT-Fatigue (“Trouble or falling/staying asleep or sleeping to much”) or PSQI (“Feeling tired or having little energy”) were excluded from analysis. The PSQI sleep efficiency scale was excluded because it is a function of the sleep duration and sleep efficiency scales. The three-item PSQI sleep dysfunction scale was also excluded due to overlap with items from the FACIT-Fatigue (“How often have you had trouble staying awake while driving, eating meals, or engaging in social activity?” and “How much of a problem has it been for you to keep up enough enthusiasm to get things done?”).

### Statistical analysis

Latent class cluster analysis was conducted to identify unobserved (latent) groups based on symptoms of fatigue, depression, and sleep problems at T1. Latent class modeling is a data-driven approach, used to classify similar objects, with respect to a set of indicators, into groups [25, 26]. Based on the responses on these indicators, the model estimates posterior probabilities of class membership. That is, an individual can have a 80% probability of belonging to class 1 and 20% probability of belonging to class 2. Indicators used to define the classes were the FACIT-Fatigue and PHQ9 items and the PSQI component scales at T1. The optimal number of classes was derived from goodness-of-fit statistics and expert opinion on clinical relevance of the classes. Bivariate residuals were assessed to check if the local independency assumption was met. When bivariate residuals remain high with increasing number of classes in the model, the local independency assumption was relaxed. The model fit was assessed by differences in log-likelihood using bootstrapped *p*-values.

Sociodemographic and clinical characteristics were compared across the classes using bivariate analysis, with chi^2^ analyses for categorical variables and analysis of variance (ANOVA) for continuous variables. A generalized version of the weighted step-three approach was used as proposed by Bolck, Croon, and Hagenaars (2004) (BCH adjustment) [27]. Means and standard errors or percentages and standard errors were reported, with the *p*-values of the Wald test.

A multinomial logistic regression analyses was conducted to assess the associations of circadian parameters with the latent classes. Sociodemographic and clinical variables were entered as covariates and backward selection was performed to ensure control of significant covariates at *p* < 0.05. Means and standard errors, and odds ratios (OR) and 95% confidence intervals (CI) were reported, with *p*-values of the Wald test.

To assess changes in class allocation from T1 to T2, latent transition analysis was conducted [28]. Because of a considerable dropout of participants at follow-up, the measurement model of the latent transition analysis was estimated on data from T1 only and subsequently compared with the observed response patterns at T2. Based on these classifications, the transition probability from T1 to T2 was estimated. In addition, transition probabilities were compared between circadian types.

Analyses were conducted with Latent GOLD version 5.1 (Statistical Innovations Inc., Belmont, MA, USA).

## Results

As previously reported, there were no meaningful or statistically significant differences between participants and non-participants of the ACCEL-Breast study for age, socio-economic status, time since diagnosis, or cancer grade [19]. Breast cancer survivors at T1 (*N* = 265) had an average age of 60 years and the majority were highly educated (trade/technical qualification or higher, 62.2%), Caucasian (92.5%), partnered (76.6%), and employed (52.1%). Most women had been diagnosed with stage I (44.5%) or II breast cancer (30.6%), and treated with surgery (98.9%), chemotherapy (50.4%), radiotherapy (66.7%), and/or hormonal therapy (74.2%) (Table 1). Participants at T2 (*N* = 168) were more often highly educated (trade/technical qualification or higher, 67.8 versus 53.0%, *p* < 0.01) compared with participants who dropped out after T1 (*N* = 102) (data not shown).

**Table 1:** Baseline characteristics of breast cancer survivors included in analysis

	Total (*N* = 265)	
Age at baseline questionnaire, M, SD	60.0	10.6
Education, *N*, %		
Did not complete high school Completed high school Trade/technical qualification University degree	42 58 92 73	15.8 21.9 34.7 27.5
Ethnicity, *N*, %		
White Other	245 20	92.5 7.5
Marital status, *N*, %		
Not married/divorced/widowed Married/de facto relationship	62 203	23.4 76.6
Employment status, *N*, %		
Not working Part-time work Full-time work	127 75 63	47.9 28.3 23.8
Comorbidities, *N*, %		
No comorbidities Only high blood pressure/cholesterol Angina, heart attack, stroke, diabetes, or other cancer	145 79 40	54.9 29.9 15.2
Cancer stage at diagnosis, *N*, %		
I II III IV Unknown	118 81 19 16 31	44.5 30.6 7.2 6.0 11.7
Surgery, *N*, %	261	98.9
Chemotherapy, *N*, %	133	50.4
Radiotherapy, *N*, %	176	66.7
Hormone therapy, *N*, %	196	74.2
Months since diagnosis at baseline questionnaire, M, SD		
	27.2	4.4
BMI at diagnosis, *N*, %		
Normal Overweight Obese	102 83 60	41.6 33.9 24.5
Smoking status at diagnosis, *N*, %		
Never Former Current	149 103 13	56.2 38.9 4.9
Menopausal status at diagnosis, *N*, %		
Premenopausal Postmenopausal	191 74	72.1 27.9

**Fig. 1:**
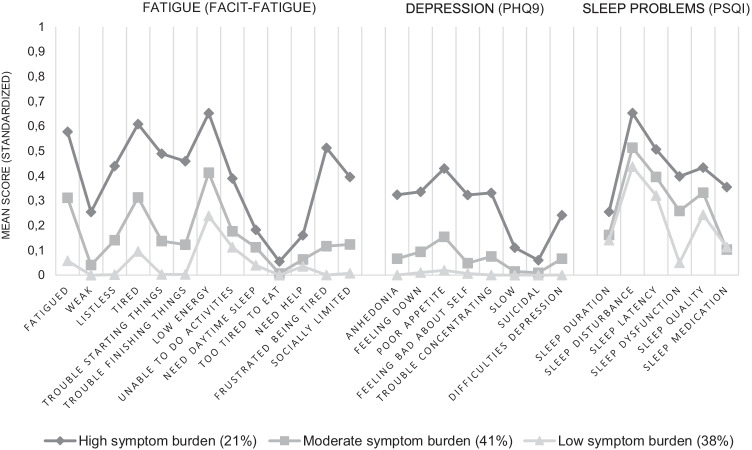
Standardized mean symptom scores by latent classes of breast cancer survivors

Using latent class analysis, we identified classes of survivors based on symptoms of fatigue (FACIT-Fatigue items), depression (PHQ9 items), and sleep problems (PSQI component scores). The local dependency assumption was relaxed for high bivariate residuals (sleep quality – sleep duration; sleep quality – sleep latency; sleep quality – sleep dysfunction). The 3-class model was selected based on the consistent Akaike’s information criterion (CAIC; Appendix Table 5) and interpretability of the classes, as additional classes were small (e.g., 5% of the sample in fourth class) and did not substantially differ from the other classes. Furthermore, an additional fourth class did not significantly improve model fit compared to the 3-class solution including bivariate residuals (-2LL diff. 14.3, *p* = 0.20). The final model included 3 classes: (1) low symptom burden (38.5%), 2) moderate symptom burden (40.7%) and high symptom burden (20.9%) (Fig. 1).

Compared to patients with low symptom burden, patients with moderate and high symptom burden were younger (58.5 and 58.2 vs. 62.7 years, *p* < 0.01), less likely to have a partner (75.5 and 63.8 vs. 84.7%, *p* = 0.02), more likely to work full-time (32.1 and 19.5 vs. 17.3%, *p* = 0.047), and were more likely to be obese (26.5 and 35.3 vs. 17.0%, *p* = 0.04) (Table 2).

**Table 2:** Sociodemographic and clinical characteristics of breast cancer survivors by latent class, based on proportional assignment

	Low symptom burden (38.5%)		Moderate symptom burden (40.7%)		High symptom burden (20.8%)		*p*
	M/%	95% CI	M/%	95% CI	M/%	95% CI	
Age at baseline questionnaire	62.7	60.7–64.8	58.5	56.4–60.5	58.2	55.5–61.0	** < 0.01**
Education							0.19
Did not complete high school	15.7	8.5–22.8	12.5	6.1–18.8	22.8	11.6–34.1	
Completed high school	26.4	17.8–35.1	19.6	11.9–27.3	18.0	7.6–28.3	
Trade/technical qualification	32.8	23.5–42.0	32.9	23.8–41.9	41.9	28.7–55.2	
University degree	25.2	16.6–33.7	35.1	25.9–44.3	17.3	7.0–27.5	
Ethnicity							0.33
Caucasian	90.2	84.3–96.1	92.2	87.1–97.4	97.1	92.6–100	
Other	9.8	3.9–15.7	7.8	2.6–12.9	3.0	0–7.4	
Marital status							**0.02**
Not married/divorced/widowed	15.3	8.2–22.4	24.5	16.2–32.8	36.2	23.3–49.1	
Married/de facto relationship	84.7	77.6–91.8	75.5	67.2–83.9	63.8	50.9–76.7	
Employment status							**0.047**
Not working	57.3	47.6–67.1	36.9	27.5–46.2	52.2	38.8–65.6	
Part-time work	25.4	16.9–33.9	31.1	22.2–39.9	28.3	16.3–40.3	
Full-time work	17.3	9.8–24.8	32.1	23.0–41.1	19.5	8.6–30.4	
Comorbidities							
No comorbidities	49.9	40.1–59.8	59.4	49.9–68.9	55.4	42.1–68.8	0.61
High blood pressure/cholesterol	34.6	25.2–43.9	24.6	16.2–33.0	31.6	19.1–44.1	
Angina, heart attack, stroke, diabetes, or other cancer	15.5	8.4–22.6	16.0	8.9–23.1	13.0	3.9–22.1	
Cancer stage at diagnosis							1.00
I	1.0	0.0–3.0	0.0	0.0–0.0	1.9	0–5.5	
II	51.6	41.7–61.4	38.8	29.4–48.2	42.7	29.4–56.0	
III	27.8	19.0–36.6	30.5	21.7–39.4	35.7	22.9–48.6	
IV	4.7	0.5–8.9	9.4	3.7–15.1	7.3	0.2–14.4	
Unknown	4.7	0.5–8.9	8.5	3.2–13.9	3.6	0–8.7	
Surgery	100.0	100–100	97.1	93.9–100.2	100.0	100–100	1.00
Chemotherapy	42.1	32.4–51.8	53.0	43.3–62.7	60.6	47.4–73.8	0.08
Radiotherapy	64.8	55.3–74.2	67.6	58.5–76.6	68.4	56.0–80.8	0.87
Hormone therapy	72.5	63.7–81.2	76.7	68.5–84.9	72.8	60.8–84.8	0.77
Months since diagnosis at baseline questionnaire	34.7	33.9–35.6	35.4	34.6–36.3	34.6	33.4–35.7	0.41
BMI at diagnosis							**0.04**
Normal	45.0	34.9–55.0	46.3	36.4–56.3	25.3	12.9–37.7	
Overweight	38.1	28.3–47.9	27.2	18.2–36.2	39.4	25.3–53.5	
Obese	17.0	9.3–24.6	26.5	17.7–35.3	35.3	21.6–49.0	
Smoking status at diagnosis							0.96
Never	58.4	48.7–68.1	56.4	46.9–66.0	51.8	38.4–65.2	
Former	36.8	27.3–46.2	39.0	29.6–48.4	42.5	29.3–55.8	
Current	4.8	0.7–8.9	4.6	0.6–8.6	5.7	0–11.9	
Menopausal status at diagnosis							0.09
Premenopausal	79.5	71.5–87.4	69.3	60.4–78.2	63.9	50.9–76.8	
Postmenopausal	20.5	12.6–28.5	30.7	21.8–39.6	36.2	23.2–49.1	

Significant *p* values (*p*<0.05) are in bold

After adjustment for covariates using backward selection (age and marital status), survivors with a late chronotype (“evening types”) were more likely to have moderate (OR = 3.38, 95% CI = 2.62–4.14, *p* < 0.01) or high (OR = 5.12, 95% CI = 4.16–6.08, *p* < 0.01) symptom burden compared to survivors with an early circadian phase (“morning types”). Furthermore, survivors with a moderate chronotype (“neither type”) were more likely to have a high symptom burden (OR = 2.11, 95% CI = 1.15–3.07, *p* < 0.05) compared to survivors with an early circadian phase (“morning type”). Survivors with a low circadian amplitude (“languid type”) or moderate circadian amplitude (“neither type”) were more likely to have moderate (OR_languid_ = 2.44, 95% CI = 1.71–3.18, *p* < 0.01; OR_neither_ = 2.02, 95% CI = 1.31–2.72, *p* < 0.01) or high (OR_languid_ = 5.56, 95% CI = 4.64–6.49, *p* < 0.01; OR_neither_ = 2.66, 95% CI = 1.72–3.60, *p* < 0.01) symptom burden compared to survivors with a high circadian amplitude (“vigorous type”) (Table 3).

**Table 3:** Multinomial logistic regression analysis of circadian parameters and symptom clusters

	Low symptom burden (38%)		Moderate symptom burden (41%)		High symptom burden (21%)	
	OR	95% CI	OR	95% CI	OR	95% CI
Chronotype						
Morning type (ref.)						
Neither type	1.00	Ref	1.73	0.74–0.99	**2.11**	1.15–3.07*
Evening type	1.00	Ref	**3.38**	2.62–4.14**	**5.12**	4.16–6.08**
Circadian amplitude						
Vigorous type (ref.)						
Neither type	1.00	Ref	**2.02**	1.31–2.72**	**2.66**	1.72–3.60**
Languid type	1.00	Ref	**2.44**	1.71–3.18**	**5.56**	4.64–6.49**
Circadian stability						
Rigid type (ref.)						
Neither type	1.00	Ref	0.66	0.00–1.38	0.40	0.00–1.33
Flexible type	1.00	Ref	0.67	0.00–1.38	0.94	0.15–1.33

Analyses were adjusted for significant covariates (age and marital status)

Significant *p* values (*p*<0.05) are in bold

^*^p < 0.05

^**^p < 0.01

Latent transition models showed that the majority of survivors in each class (80.3% low symptom burden; 58.6% moderate symptom burden; 63.6% high symptom burden) remained in their class at T2. However, 29.6% of survivors with moderate symptom burden and 34.5% of survivors with high symptom burden at T1 moved to low and moderate symptom burden classes respectively. Furthermore, 18.4% of survivors with low symptom burden and 11.9% of survivors with moderate symptom burden moved to moderate and high symptom burden classes respectively (Table 4).

**Table 4:** Transitions between symptom clusters from T1 (*N* = 265) to T2 (*N* = 169)

	T2 low symptom burden (55.3%)	T2 moderate symptom burden (32.8%)	T2 high symptom burden (11.9%)
T1 low symptom burden (38.5%)	80.3%	18.4%	1.3%
T1 medium symptom burden (40.7%)	29.6%	58.6%	11.9%
T1 high symptom burden (20.8%)	2.0%	34.5%	63.6%

The percentages show the proportions of patients that moved from low, medium, or high symptom burden classes at T1 to low, medium, or high symptom burden classes at T2. e.g., 29.6% of the survivors with a medium symptom burden at T1 moved to a low symptom burden at T2

A higher proportion of evening types than morning types had persistent symptom burden (persistent high 75 vs. 36%; persistent moderate 65 vs. 49%), and a lower proportion moved to a lower symptom burden class (high to moderate 24 vs. 59%; moderate to low 26 vs. 34%) (Appendix Table 6). A higher proportion of vigorous types than languid types had low symptom burden at T1, and a higher proportion of languid types than vigorous types had persistent low symptom burden at T2 (92 vs. 74%) (Appendix Table 7). Furthermore, transition probabilities were similar across circadian stability types except for rigid compared with flexible types with moderate symptom burden at T1, who showed a small trend towards the low symptom burden class (38 vs. 19%; Appendix Table 8).

## Discussion

In this sample of breast cancer survivors, a high burden of symptoms of fatigue, depression, and sleep problems clustered in 21% of the population, while another 41% showed moderate levels of these symptoms, at 2–3 years after diagnosis. A late chronotype (“evening type”) and low circadian amplitude (“languid type”) after diagnosis were associated with a higher symptom burden after 2–3 years. The majority of survivors had persistent symptom burden after 6–8 years, with eveningness associated with lower symptom recovery.

The clustering of fatigue, depression, and sleep problems is consistent with previous studies in breast cancer survivors and other cancer populations [4]. However, sleep problems were prevalent across all identified symptom burden classes, with a PSQI global score above the clinical cut-off of 5 in each class [24]. Therefore, sleep problems were prevalent but did not cluster with depression and fatigue symptoms in the low symptom burden class. Interestingly, while previous results from our baseline data (BCEES) showed that survivors with a low circadian stability (“flexible type”) had a lower sleep duration [18], flexible types were not more likely to have a higher symptom burden in the current study. A possible explanation is that flexible types may cope better with sleep deprivation and be less vulnerable to poor sleep quality [13] and clustering with other symptoms. Associations with low circadian amplitude (“languid type”, i.e., the inability to overcome drowsiness) remain inconclusive; its similarity to fatigue meant that languidness was inevitably associated with high symptom burden. The similarity to fatigue may also explain why latent transition models suggested that languid types were more likely to experience persistent levels of symptom burden compared to vigorous types, and suggests that interventions to decrease overall symptom burden may be particularly beneficial for languid types.

Similar to studies in non-cancer populations [14, 15, 18], eveningness was associated with higher symptom burden. This has previously been explained by a dysregulation of the hypothalamic–pituitary–adrenal (HPA) axis in evening types, as indicated by a decreased cortisol awakening response [12, 29]. According to the social jet lag theory, evening types show more misalignment of biological clock and social schedules, resulting in insufficient sleep, more fatigue, and more mental exhaustion [30]. However, symptoms of fatigue, depression, and sleep problems may in turn trigger dysregulation of the HPA axis [31]. Therefore, the causal relationships between circadian preference and these symptoms remained largely unclear using cross-sectional designs [14, 15, 18]. Albeit not statistically significant (due to small numbers), our transition models provide some evidence for eveningness being a risk factor of high symptom burden, a finding that merits further investigation.

While breast cancer treatments such as chemotherapy and radiotherapy have previously been found to trigger circadian dysregulation [9], neither treatment was a significant covariate in the relationship between circadian parameters and symptom burden in our analysis. Therefore, circadian preference in breast cancer survivors may be similar to that of the general population. Yet, even when circadian parameters are considered stable characteristics not influenced by the cancer and treatments, the identification of circadian types as potential risk factors may support targeted interventions to reduce long-term symptom burden.

### Limitations

Although the three classes with different levels of overall symptom burden may provide evidence for a common, underlying clustering, our small sample size limited the possibility to identify additional subgroups of patients with more unique clustering of symptoms (i.e., depression symptoms but no fatigue or sleep problems). Importantly, symptom burden was measured only at follow-up, whereas circadian parameters were measured at diagnosis. Therefore, it is possible that symptom burden was already present at diagnosis and is not causally associated with circadian parameters. While our transition models provide some evidence for a causal relationship, further longitudinal or intervention research is warranted.

Only a selection of participants (i.e., the 600 most recently diagnosed) from the baseline study (BCEES) were selected for participation in the current study. Although there were no meaningful or statistical differences in sociodemographic or clinical variables between participants and non-participants at T1 [19], our study population was primarily Caucasian and well educated, particularly so for our sample at T2 due to selective drop-out. Although drop-out in survivorship research is common [32], our sample size and loss-to-follow-up (38%) resulted in limited statistical power of the transition models. Therefore, transition data were descriptive, not statistically tested, and should be interpreted with caution.

Furthermore, the overall symptom burden in our sample is relatively low compared to other cancer populations [22, 33] and morningness is relatively high [20, 34]. As a result, the tertile with highest eveningness in our sample may be misclassified as “evening type”, when compared to cutoffs that were validated in a student sample [20] or working population [34].

### Future directions

This is the first study examining the overall burden of fatigue, depression, and sleep problems among cancer survivors in relation to circadian rhythm parameters. Whereas the types of cluster analysis that have traditionally been used in symptom cluster research (e.g., principal components analysis and common factor analysis using deterministic cluster assignment [5]) use deterministic cluster allocation, latent class analysis is a probabilistic statistical technique that accounts for uncertainty of cluster assignment and therefore prevents bias in our circadian parameter estimates. Furthermore, latent transition models provided the unique opportunity to assess transitions in symptom clustering over time. Despite the limitations of our small sample size, this study provides valuable new insights into the long-term associations of chronotype, circadian amplitude, and circadian stability with symptom burden in breast cancer survivors.

While associations with languidness remained inconclusive, eveningness was identified as a potential risk factor for long-term, co-occurring symptoms of fatigue, depression, and sleep problems in breast cancer survivors. Innate chronotype is largely influenced by non-modifiable factors including genetics and age; however, if the associations seen in our study are due to disruption of circadian rhythm in evening type women, it may be worth investigating interventions such as bright light exposure [16] and melatonin supplements [17] during or after breast cancer treatment which have shown promising effects on circadian re-alignment. Therefore, psycho-oncological or pharmaceutical therapies to re-align survivors’ chronotype and circadian amplitude could provide important directions to targeted interventions in evening types and may subsequently reduce long-term symptom burden. In future research, objective methods such as actigraphy and hormones including (24-h) cortisol and melatonin could provide more detailed information on chronotype for personalized therapies for re-alignment of chronotype and circadian amplitude [35, 36].

In conclusion, breast cancer survivors who are evening types may be at higher risk of accumulating symptoms of fatigue, depression, and sleep problems and may additionally be less likely to recover from these symptoms. Chronotype may be a novel target in interventions aimed at alleviating symptom burden among breast cancer survivors.

### Electronic supplementary material

Below is the link to the electronic supplementary material.

Supplementary file 1 Flow chart of patients included in the current analysis (PDF 73 KB)

## Data Availability

The dataset cannot be shared due to ethical approval constraints.

## Appendix

**Table 5:** Fit indexes of latent class analyses

Model	LL	BIC_LL_	AIC_LL_	AIC^3^_LL_	CAIC_LL_	L^2^	Bootstrap *P*-value	-2 LL Diff	-2 LL Diff. *P*-value
1-Class	− 6142.6200	12,787.4157	12,465.2400	12,555.2400	12,877.4157	9668.4609	0.19	N/A	N/A
2-Class	− 5348.2665	11,354.9411	10,932.5330	11,050.5330	11,472.9411	8079.7539	0.32	1588.70	< 0.01
3-Class	− 5095.6612	11,005.9629	10,483.3224	10,629.3224	**11,151.9629**	7574.5433	0.36	505.21	< 0.01
4-Class	− 5006.2977	**10,983.4684**	10,360.5954	10,534.5954	11,157.4684	7395.8163	0.32	178.73	< 0.01
5-Class	− 4940.4170	11,007.9393	10,284.8339	10,486.8339	11,209.9393	7264.0548	0.27	131.76	< 0.01
6-Class	− 4870.8717	11,025.0813	**10,201.7435**	**10,431.7435**	11,255.0813	7124.9644	0.30	139.09	< 0.01

*BIC* Bayes information criterion, *AIC* Aikake’s information criterion, *CAIC* consistent Aikake’s information criterion, *LL* log-likelihood

Lowest values of are in bold

**Table 6:** Transitions in class allocation from T1 to T2, by chronotype

	T2 low symptom burden	T2 moderate symptom burden	T2 high symptom burden
Morning type			
T1 low symptom burden	77.8%	21.2%	1.0%
T1 moderate symptom burden	34.4%	49.0%	16.6%
T1 high symptom burden	4.4%	59.4%	36.3%
Neither type			
T1 low symptom burden	81.1%	17.5%	1.4%
T1 moderate symptom burden	30.2%	57.3%	12.4%
T1 high symptom burden	2.6%	40.8%	56.6%
Evening type			
T1 low symptom burden	83.6%	14.3%	2.1%
T1 moderate symptom burden	25.8%	65.2%	9.1%
T1 high symptom burden	1.3%	23.8%	74.9%

The percentages show the proportions of patients that moved from low, medium, or high symptom burden classes at T1 to low, medium, or high symptom burden classes at T2

**Table 7:** Transitions in class allocation from T1 to T2, by circadian amplitude

	T2 low symptom burden	T2 moderate symptom burden	T2 high symptom burden
Vigorous type			
T1 low symptom burden	73.9%	23.8%	2.3%
T1 moderate symptom burden	38.2%	46.5%	15.4%
T1 high symptom burden	10.4%	24.3%	65.3%
Neither type			
T1 low symptom burden	85.1%	14.4%	0.5%
T1 moderate symptom burden	28.6%	59.8%	11.7%
T1 high symptom burden	1.1%	32.4%	66.5%
Languid type			
T1 low symptom burden	91.7%	8.2%	0.1%
T1 moderate symptom burden	19.9%	71.8%	8.3%
T1 high symptom burden	0.1%	38.9%	61.0%

The percentages show the proportions of patients that moved from low, medium, or high symptom burden classes at T1 to low, medium, or high symptom burden classes at T2

**Table 8:** Transitions in class allocation from T1 to T2, by circadian stability

	T2 low symptom burden	T2 moderate symptom burden	T2 high symptom burden
Rigid type			
T1 low symptom burden	79.6%	20.4%	0.0%
T1 moderate symptom burden	37.9%	55.8%	6.4%
T1 high symptom burden	5.6%	31.8%	62.7%
Neither type			
T1 low symptom burden	81.4%	18.1%	0.5%
T1 moderate symptom burden	27.9%	60.3%	11.8%
T1 high symptom burden	1.0%	34.7%	64.3%
Flexible types			
T1 low symptom burden	79.7%	15.4%	4.9%
T1 moderate symptom burden	19.1%	60.7%	20.2%
T1 high symptom burden	0.2%	36.3%	63.5%

The percentages show the proportions of patients that moved from low, medium, or high symptom burden classes at T1 to low, medium, or high symptom burden classes at T2
