# Supplementary figures and images for: The association of circadian parameters and the clustering of fatigue, depression, and sleep problems in breast cancer survivors: a latent class analysis
Source: J Cancer Surviv. 2022 Mar 23;17(5):1405–15. doi: 10.1007/s11764-022-01189-w (PMC10442261; doi:10.1007/s11764-022-01189-w)

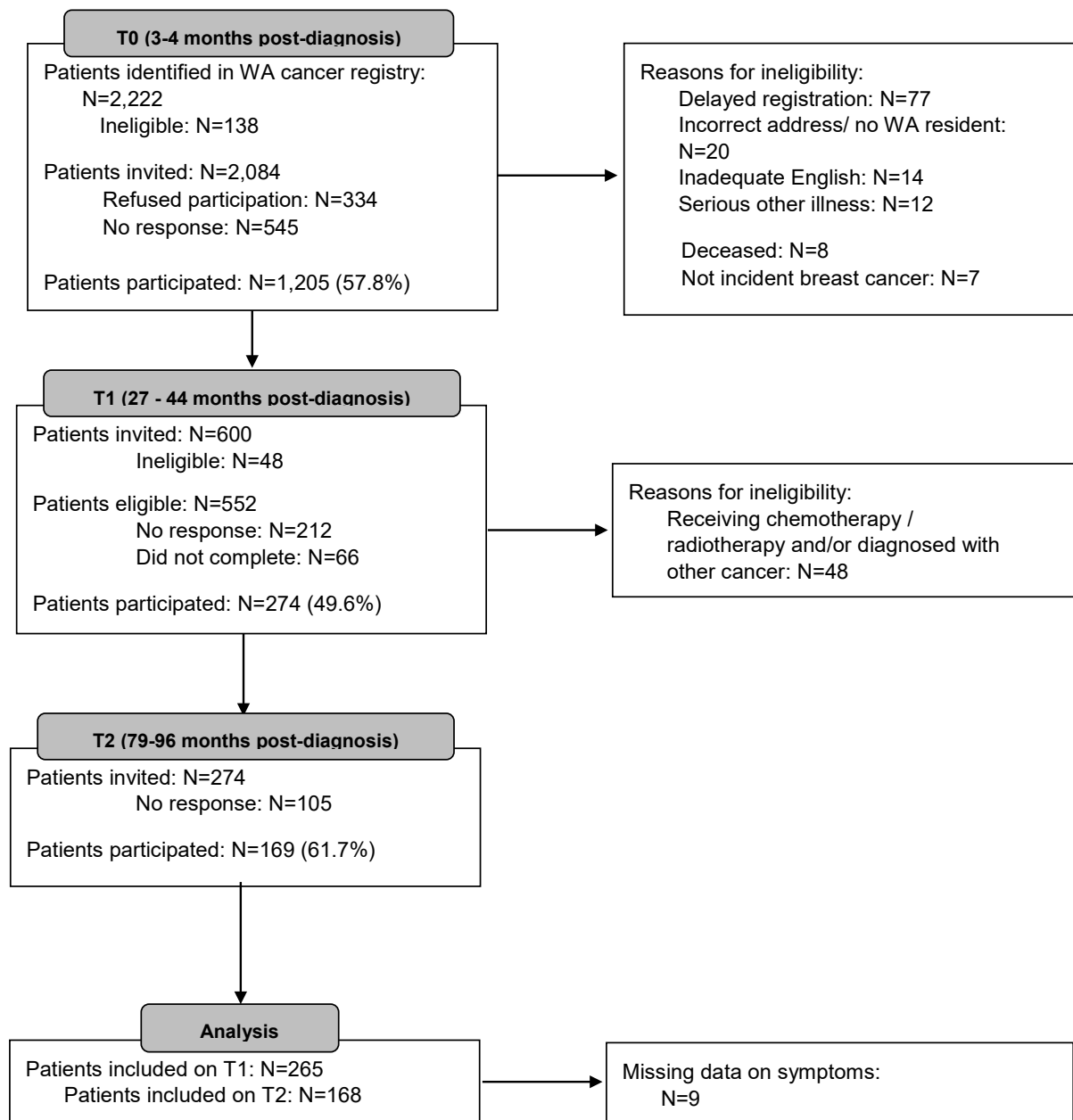

Supplement: Supplementary file 1 — Flow chart of patients included in the current analysis (PDF 73 KB) [file 11764_2022_1189_MOESM1_ESM.pdf]
